# Supplementary material for: Polyphenolics with Strong Antioxidant Activity from Acacia nilotica Ameliorate Some Biochemical Signs of Arsenic-Induced Neurotoxicity and Oxidative Stress in Mice
Source: Molecules. 2022 Feb 3;27(3):1037. doi: 10.3390/molecules27031037 (PMC8840196; doi:10.3390/molecules27031037)
Supplement: Supplementary file 1 [file molecules-27-01037-s001.zip › molecules-1560802-supplementary.pdf]

Supplementary Materials

Polyphenolics with Strong Antioxidant Activity from *Acacia nilotica* Ameliorate Some Biochemical Signs of Arsenic-Induced Neurotoxicity and Oxidative Stress in Mice

Tahira Foyzun [1], Abdullah Al Mahmud [2], Md. Salim Ahammed [2], Md. Imran Nur Manik [3], Md. Kamrul Hasan [4], KM Monirul Islam [2], Simin Sobnom Lopa [2], Md. Yusuf Al-Amin [2], Kushal Biswas [5], Mst. Rejina Afrin [5], AHM Khurshid Alam [2] and Golam Sadik [2*]

[1] Department of Pharmacy, Southeast University, Dhaka 1212, Bangladesh; tahirafoyzun87@gmail.com
[2] Department of Pharmacy, University of Rajshahi, Rajshahi 6205, Bangladesh; littleru21@gmail.com (A.A.M.); salimahamad2017@gmail.com (M.S.A); moniferdous@yahoo.com (K.M.I.); siminshabnam@yahoo.com (S.S.L.); yusufrupharma@yahoo.com (M.Y.A.-A.); khurshid.jaist@gmail.com (A.K.A.)
[3] Department of Pharmacy, Northern University Bangladesh, Dhaka 1205, Bangladesh; imran.md39@gmail.com
[4] Department of Pharmacy, Comilla University, Kotbari, Cumilla 3506, Bangladesh; kh.shikhon22@gmail.com
[5] Department of Pharmacy, East West University, Dhaka 1212, Bangladesh; kushal71@outlook.com (K.B.); rejinaafrin@ymail.com (M.R.A.)
* Correspondence: gsadik2@yahoo.com

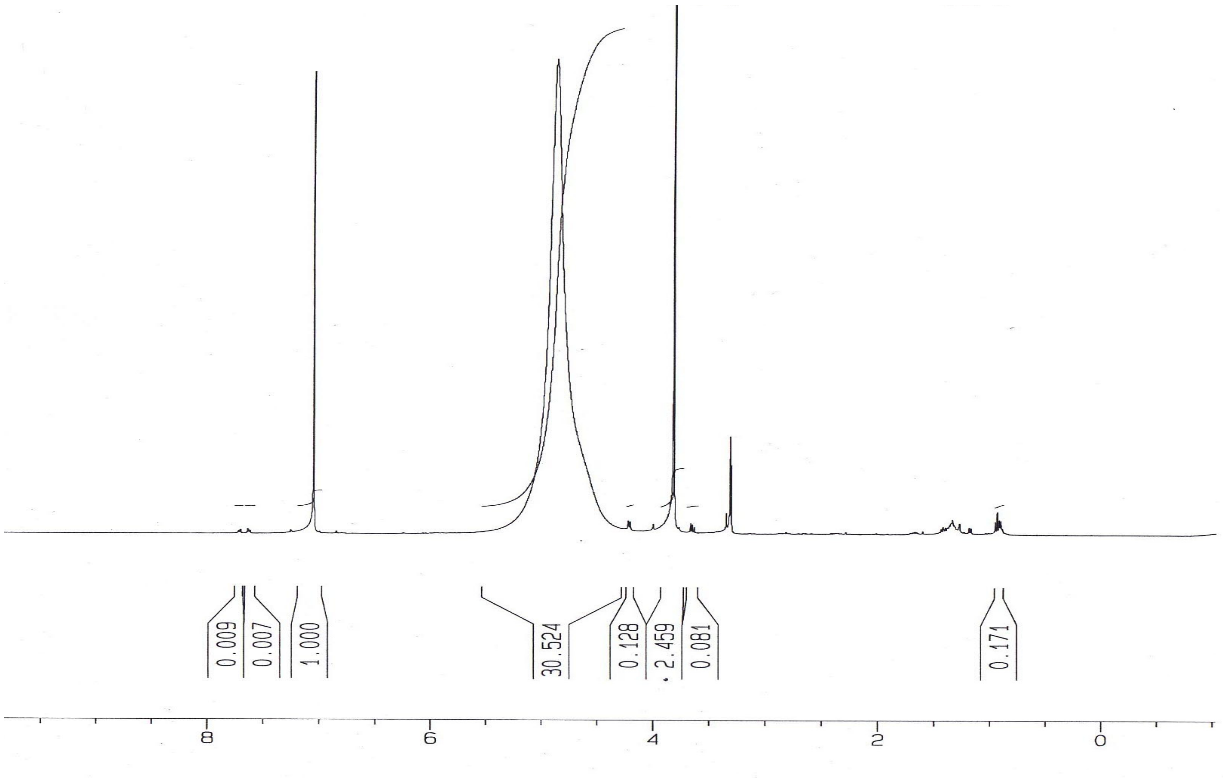

Figure S1. ^{1}H NMR (400 MHz, CD_3OD) spectrum of compound **1**.

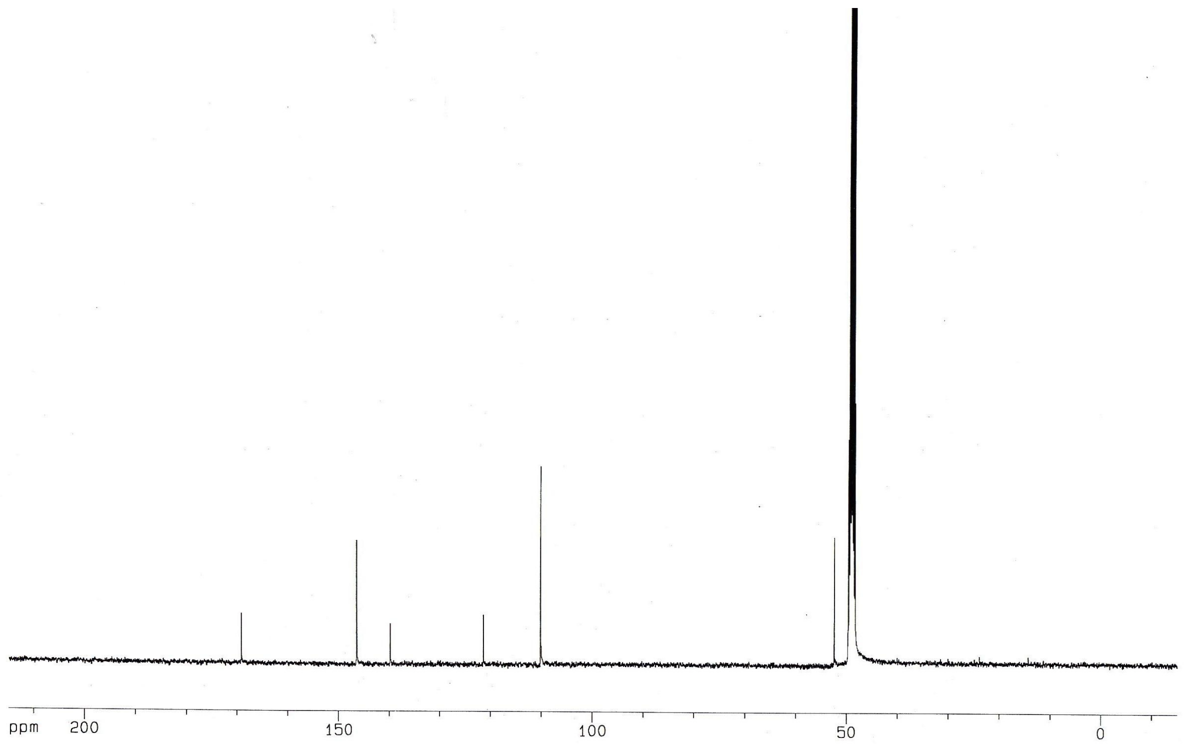

Figure S2. ^{13}C NMR (100 MHz, CD_3OD) spectrum of compound **1**.

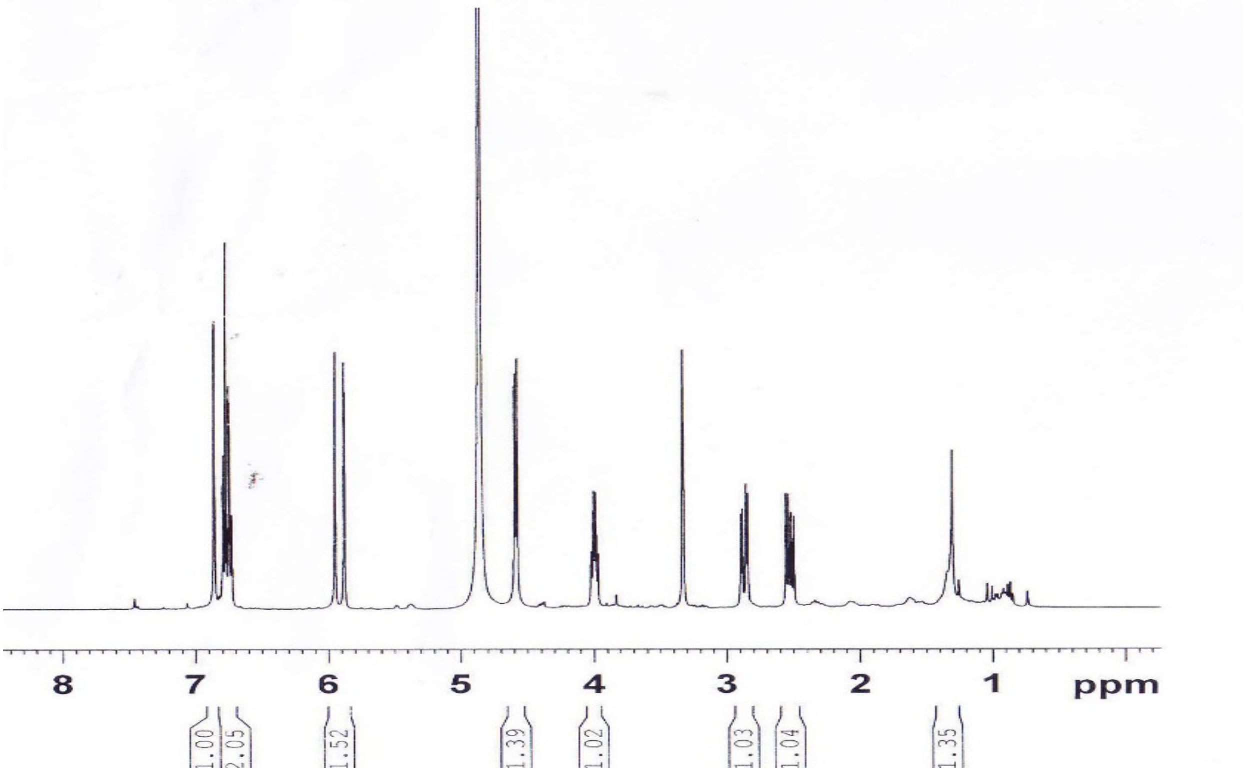

Figure S3. ^{1}H NMR (400 MHz, CD_3OD) spectrum of compound **2**.

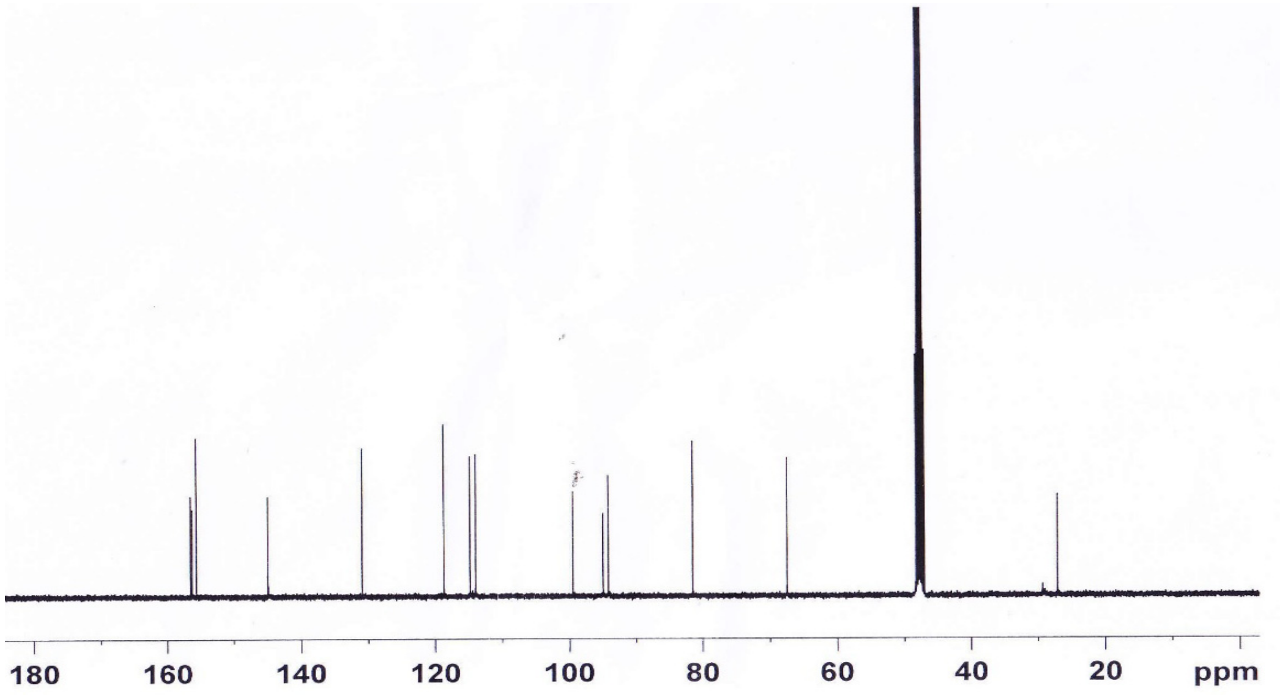

Figure S4. ^{13}C NMR (100 MHz, CD_3OD) spectrum of compound **2**.

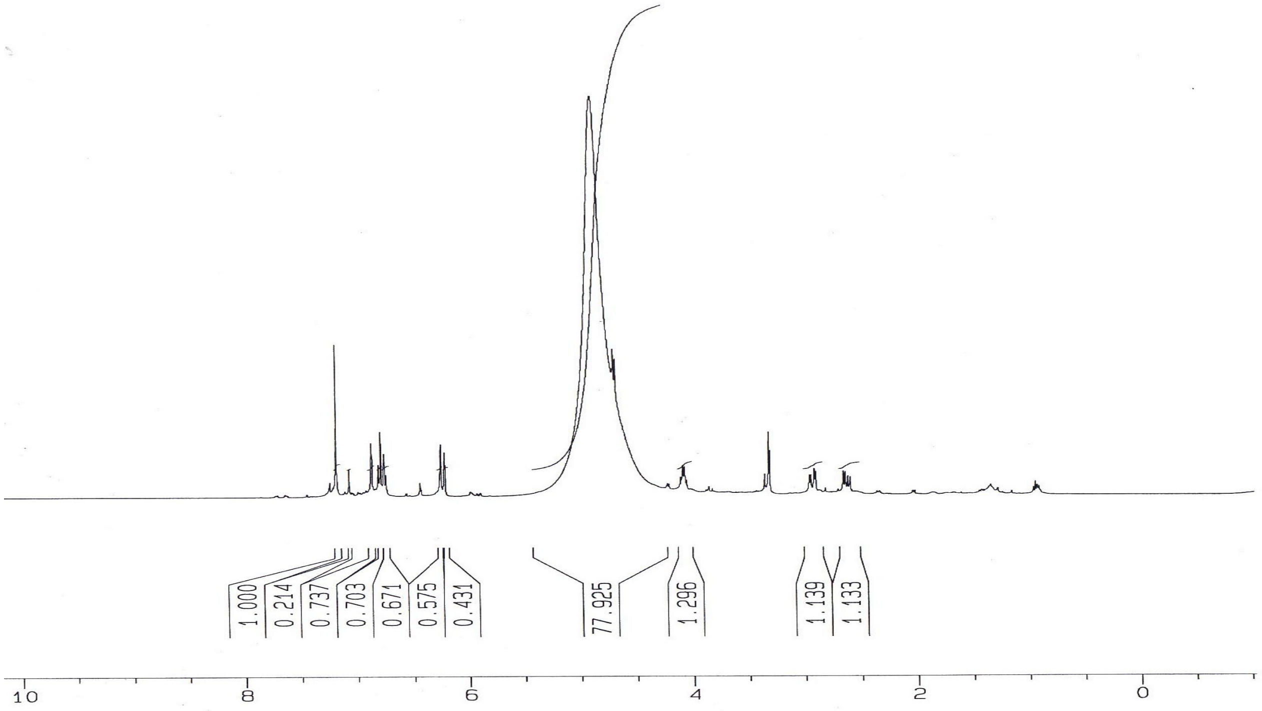

Figure S5. ^1H NMR (400 MHz, CD_3OD) spectrum of compound **3**.
